# Effects of Posture and Walking on Tibial Vascular Hemodynamics Before and After 14 Days of Head‐Down Bed Rest

**DOI:** 10.1002/jbm4.10756

**Published:** 2023-05-02

**Authors:** Eric T Hedge, Laurence Vico, Richard L Hughson, Carmelo J Mastrandrea

**Affiliations:** ^1^ Schlegel‐UW Research Institute for Aging Waterloo Canada; ^2^ Department of Kinesiology and Health Sciences University of Waterloo Waterloo Canada; ^3^ U1059 INSERM—SAINBIOSE (Santé Ingéniérie Biologie St‐Etienne) Campus Santé Innovation Université Jean Monnet Saint‐Priest‐en‐Jarez France

**Keywords:** HEAD‐DOWN BED REST, NEAR INFRARED SPECTROSCOPY (NIRS), SKELETAL VASCULAR SUPPLY, TIBIAL HEMODYNAMICS

## Abstract

Human skeletal hemodynamics remain understudied. Neither assessments in weight‐bearing bones during walking nor following periods of immobility exist, despite knowledge of altered nutrient‐artery characteristics after short‐duration unloading in rodents. We studied 12 older adults (8 females, aged 59 ± 3 years) who participated in ambulatory near‐infrared spectroscopy (NIRS) assessments of tibial hemodynamics before (PRE) and after (POST) 14 days of head‐down bed rest (HDBR), with most performing daily resistance and aerobic exercise countermeasures during HDBR. Continual simultaneous NIRS recordings were acquired over the proximal anteromedial tibial prominence of the right lower leg and ipsilateral lateral head of the gastrocnemius muscle during supine rest, walking, and standing. During 10 minutes of walking, desaturation kinetics in the tibia were slower (time to 95% nadir values 125.4 ± 56.8 s versus 55.0 ± 30.1 s, *p* = 0.0014). Tibial tissue saturation index (TSI) immediately fell (−9.9 ± 4.55) and did not completely recover by the end of 10 minutes of walking (−7.4 ± 6.7%, *p* = 0.027). Upon standing, total hemoglobin (tHb) kinetics were faster in the tibia (*p* < 0.0001), whereas HDBR resulted in faster oxygenated hemoglogin (O_2_Hb) kinetics in both tissues (*p* = 0.039). After the walk‐to‐stand transition, changes in O_2_Hb (*p* = 0.0022) and tHb (*p* = 0.0047) were attenuated in the tibia alone after bed rest. Comparisons of NIRS‐derived variables during ambulation and changes in posture revealed potentially deleterious adaptations of feed vessels after HDBR. We identify important and novel tibial hemodynamics in humans during ambulation before and after bed rest, necessitating further investigation. © 2023 The Authors. *JBMR Plus* published by Wiley Periodicals LLC on behalf of American Society for Bone and Mineral Research.

## Introduction

Skeletal losses during aging,^(^ ^1^, ^2^ ^)^ bed rest,^(^ ^3^, ^4^ ^)^ and accelerated aging‐like effects of spaceflight^(^ ^5^ ^)^ are all well described. Reduced mechanical stimulation of the bone leads to a change in metabolic set point and bone losses, a mechanism originally coined as the “mechanostat,”^(^ ^6^ ^)^ which subsequently expanded to also include nonmechanical factors.^(^ ^7^, ^8^ ^)^ Predisposition to osteoporosis and fracture, with consequential morbidity and mortality, represents significant risk to all sedentary individuals. This is especially true for those with concomitant dietary and metabolic changes, such as hospital in‐patients ^(^ ^9^ ^)^ and older adults in care settings.^(^ ^10^ ^)^

The relationships between bone metabolism and its microvasculature are well documented.^(^ ^11^, ^12^ ^)^ There are close links between osteoprogenitors and specific microvascular beds within the bone itself,^(^ ^13^ ^)^ and both exhibit simultaneous and dynamic responses to resorption stimuli in rodents.^(^ ^14^ ^)^ Larger skeletal vessels and their links to bone health have also been identified in humans, with age‐related reductions to lower limb bone perfusion^(^ ^15^ ^)^ and reduced perfusion indices in femur of older individuals with osteoporosis.^(^ ^16^ ^)^ In rodent models, changes to skeletal microvasculature include the identification of an altered vascular supply to long bones after hindlimb unloading, where both reductions to the femoral principal nutrient vessel diameter^(^ ^17^ ^)^ and attenuated vasodilatory ability^(^ ^18^ ^)^ were noted. This is of particular importance given that the vast majority of a bone's blood supply comes from this principal vessel.^(^ ^12^ ^)^ It may be that similar changes occur to arterial vessels supplying the bones of the lower body in humans during weightlessness or periods of sedentary behavior and may lead to detrimental changes to bone architecture. However, no assessments of human bone during exposure to these environments exist, necessitating further research.

Assessment of skeletal hemodynamics remains understudied, primarily attributable to the risk and discomfort of required invasive sampling. Near infrared spectroscopy (NIRS) allows for noninvasive determination of tissue saturation index (TSI), a reliable measure of tissue oxygenation,^(^ ^19^ ^)^ in addition to concentrations of oxygenated (O_2_Hb), deoxygenated (HHb), and total (tHb) hemoglobin in the tissues below the skin.^(^ ^20^ ^)^ NIRS can be applied to the medial tibia, a site with no overlying muscle and minimal adipose between the skin and anterior tibial cortex, to monitor changes in oxygenation with high levels of repeatability.^(^ ^21^ ^)^ To date, investigations in the tibia have confirmed that NIRS signals were sensitive to changes in bone blood flow with tilt or reperfusion after ischemic occlusion.^(^ ^21^, ^22^, ^23^, ^24^, ^25^ ^)^ NIRS identified changes in O_2_Hb and HHb in the tibia during and after rowing that recruited primarily upper leg muscles of able‐bodied participants and functional electrical stimulation of quadriceps and hamstring muscles in spinal cord–injured participants.^(^ ^26^ ^)^ However, tibial hemodynamics during walking or normal daily activity remain unassessed.

In our daily lives, upright posture with walking and standing affect lower limb perfusion as sympathetic activation constricts the vessels of the lower limb compared with supine posture.^(^ ^27^ ^)^ With muscular contractions, release of vasodilator metabolites to reduce vascular resistance and activation of the muscle pump to enhance perfusion pressure gradient combine to adjust perfusion to the metabolic demand.^(^ ^28^, ^29^ ^)^ Bone perfusion during physical exercise is unknown. Recent evidence suggests that the arterial supply of the tibia might be responsive to sympathetic vasoconstriction,^(^ ^30^ ^)^ increasing vascular resistance and reducing flow. On the other hand, arterial pressure increases in upright posture, while the impact of the muscle pump acting on vessels contained within the rigid bones is unknown, leaving questions about the perfusion pressure gradient across the tibia. NIRS can provide insights into the combined effects of posture and muscular contraction on bone tissue where metabolic rate would not change, and changes in oxygenation will be related to perfusion of the bone.

Bone tissue is sensitive to changes in activity patterns, and its perfusion might be influenced by protracted physical inactivity, such as with injury, illness, or spaceflight. We had an opportunity to assess physiological deconditioning in older adults (55‐65 years of age) during a 14‐day 6° head‐down bed rest (HDBR) campaign. As part of this study, we performed ambulatory and postural NIRS assessments before and after HDBR to investigate possible HDBR‐induced changes to tibial hemodynamics. In this first study to examine the oxygenation of the tibia during a transition from supine posture to upright walking and standing, we hypothesized that TSI would drop in the tibia with walking and have a slower time course than gastrocnemius muscle, as functional hyperemia in muscle overcomes the vasoconstriction with upright posture. We hypothesized the skeletal muscle pump that generates increased perfusion pressure gradient across the contracting muscles will enhance venous outflow from the bone so that tHb in the tibia will increase on cessation of walking. The effects of 14‐day HDBR are hypothesized to appear as greater reductions in TSI and a smaller amplitude of increase in tHb on standing, reflecting altered feed vessel characteristics and potential cardiovascular deconditioning.

## Materials and Methods

### Study protocol

Twelve healthy older adults (8 females; aged 59 ± 3 years; weight 65.4 ± 12.1 kg; height 164 ± 7 cm) participated in the present investigation of bone hemodynamics before and after 14 days of 6° HDBR. All participants were healthy nonsmokers, who habitually performed a minimum of 2.5 hours of moderate‐to‐vigorous exercise per week. Female participants were at least 1 year postmenopausal with serum follicle‐stimulating hormone greater than 30 IU/L. Participants were admitted for a total of 26 days at McGill University Health Centre, comprising a 5‐day pre‐bed rest baseline data collection period, 14‐day HDBR period, and 7‐day post‐bed rest recovery period. During the pre‐bed rest and post‐bed rest periods, participants were ambulatory and free to perform normal daily activities of living. During HDBR, participants remained in a strict and supervised 6° head‐down position continually, including while showering, toileting, and eating. Participants were randomly allocated into either the control or exercise arm of the study. Those in the exercise arm performed daily exercise activities in the head‐down position throughout HDBR, including high‐intensity interval and aerobic cycling, and upper and lower body resistance training, as previously described,^(^ ^31^, ^32^ ^)^ whereas those in the control arm received daily passive physiotherapy. Blood was drawn for a full blood count before bed rest and on recovery day 6. The study was performed in accordance with the declaration of Helsinki, following ethical approval from the McGill University Research Ethics Board (IRB00010120) and the University of Waterloo Research Ethics Board (ORE# 40420), and was registered as a clinical trial (NCT04964999).

### Testing protocol

**Fig. 1:**
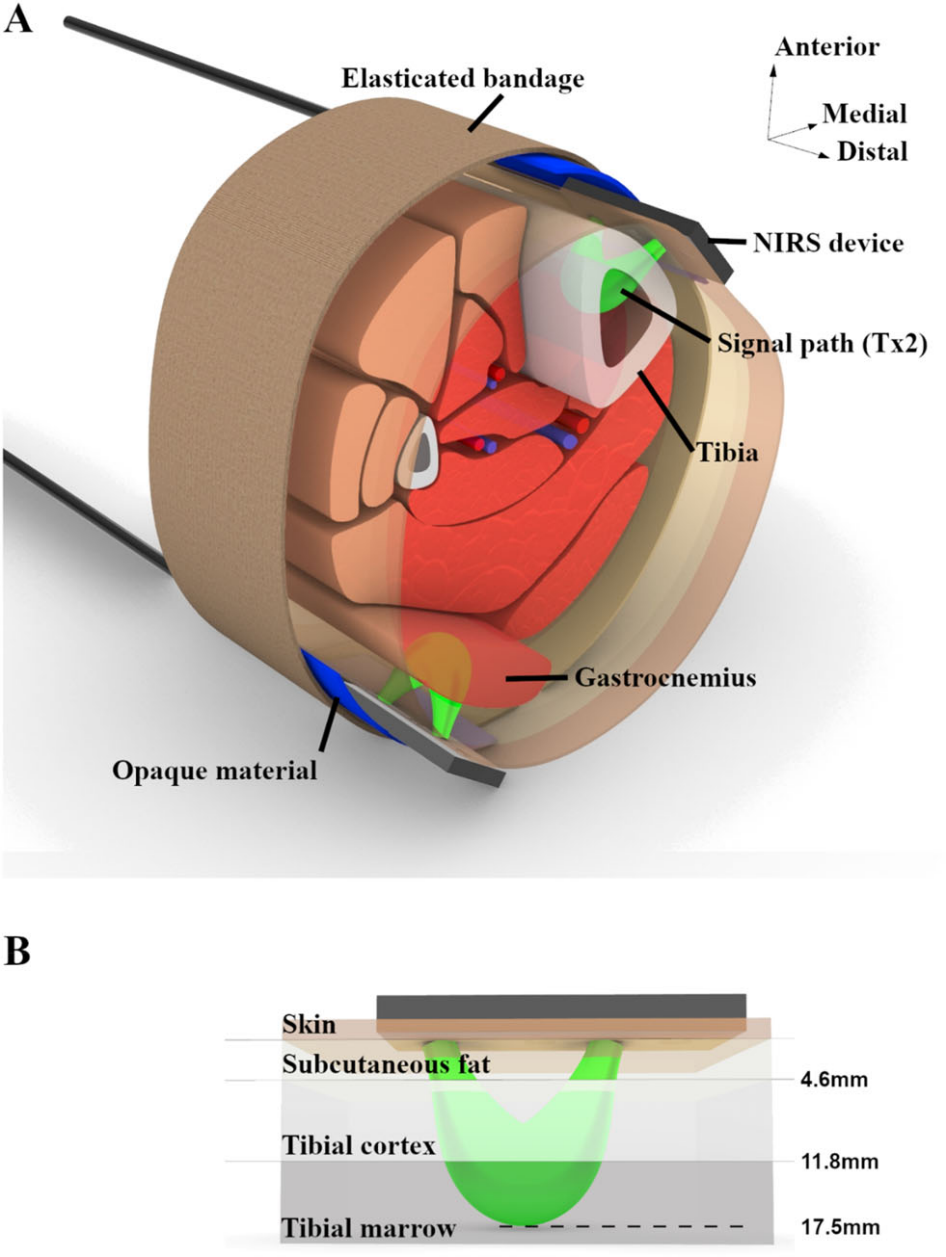
Representation of equipment setup on the right leg (*A*) with illustrative representation of the signal path through the underlying tibia (*B*). Near‐infrared spectroscopy (NIRS) devices were located immediately above the superficial tibia on the anteromedial lower limb and over the lateral head of the gastrocnemius muscle bulk in the right lower leg. Devices were covered with an opaque material before being wrapped with elasticated Tensor bandaging. Signals from the middle transmitter (Tx2) on the device were used during analysis of these data, allowing for investigation of tibial hemodynamics. Average depths of transitions between soft tissue and superficial tibial cortex, between tibial cortex and tibial marrow, and of maximal signal depth are also provided.

Testing was performed on day 3 of the pre‐bed rest baseline data collection period (PRE) and on day 5 of the post‐bed rest recovery period (POST). All tests were performed at least 2 hours post‐prandial on days in which participants refrained from consuming caffeine. Immediately before PRE data collection, longitudinal and transverse anteromedial lower leg B‐mode ultrasound images of the superficial tibia were acquired using a 17 MHz linear‐T probe (Sonoscanner, Ivry‐sur‐Seine, France). Participants were instrumented in the supine position with the equipment setup as shown in Figure 1. A continuous‐wave NIRS device (Portalite Artinis Medical Systems, Einsteinweg, Netherlands) was placed on the right anteromedial lower leg parallel to the tibia at a position identified with ultrasound to be directly above the midline of the superficial tibia (differential pathlength factor: 7, k: 1.5, h: 5e^−4^, wavelengths of 853 and 762 nm). This distance was approximately 6 cm below the knee, overlying the most superficial region of the tibia identified with ultrasound. A second similar NIRS device was placed over the lateral gastrocnemius muscle of the same leg (differential pathlength factor: 5.24, k: 1.63, h: 5.5e^−4^, wavelengths of 845 and 759 nm). The k and h represent constants required to estimate tissue scatter coefficients of the NIRS signals. These values were extrapolated from data previously published on tibial NIRS assessments in the same location we chose to measure hemodynamics in our study.^(^ ^26^ ^)^ Positions of both devices were documented to ensure consistent placement between PRE and POST collections. The devices were then covered with flexible opaque material and wrapped with elasticated tensor bandaging (3M, Saint Paul, MN, USA). To ensure the applied pressure from the bandaging onto the NIRS devices and the lower leg were similar between sessions, individual tensor bandaging was used for each participant, with marks made on the band during PRE collections to allow for reproducible placement and application pressure at the POST time point.

**Fig. 2:**
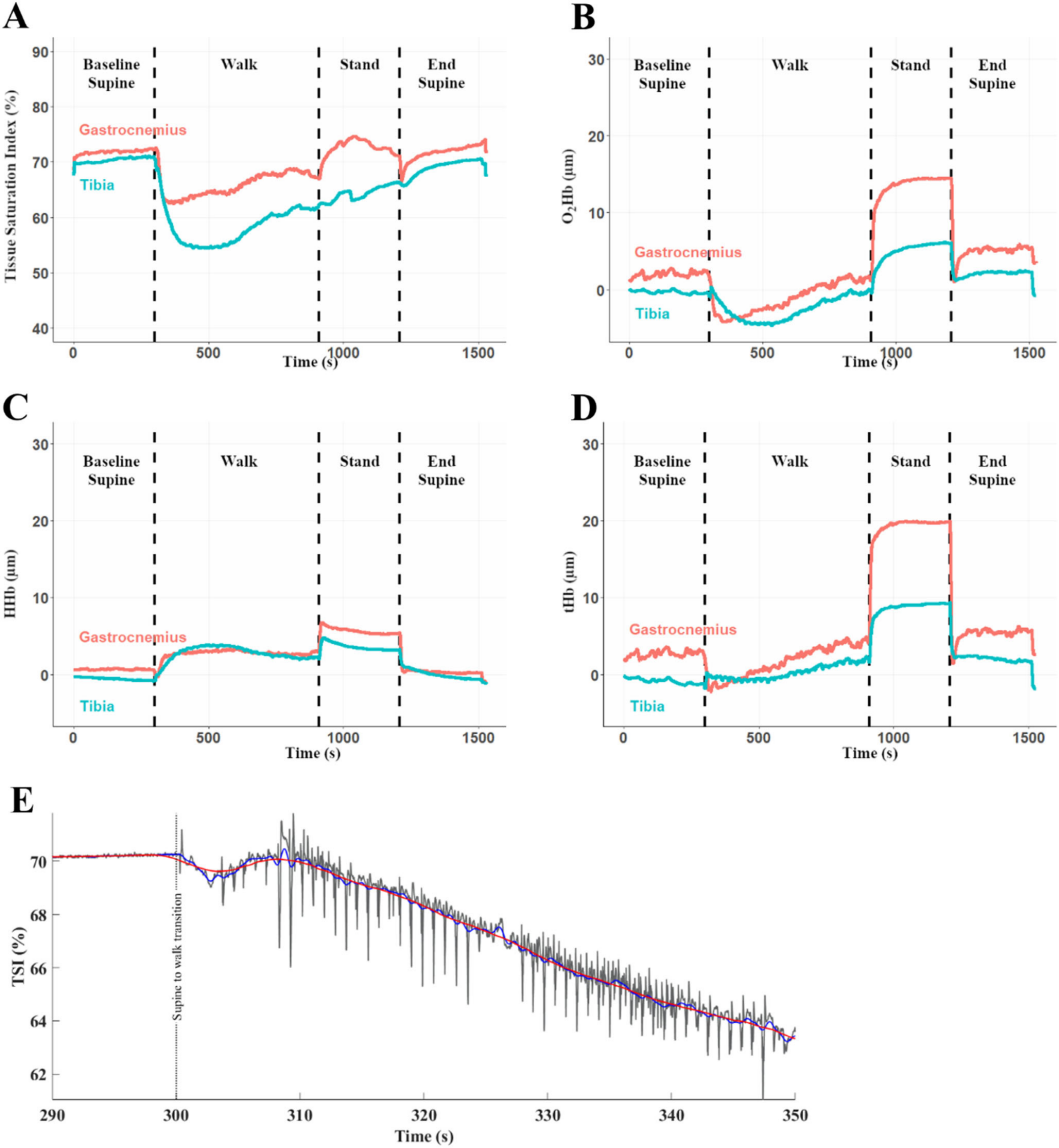
(*A–D*) Extracted, processed time series data from synchronized near‐infrared spectroscopy (NIRS) devices during one session for the tibia (blue) and gastrocnemius muscle (red). Dashed lines represent transitions between postures as described in the figure. Data presented for tissue saturation index (TSI) (*A*), oxygenated hemoglobin (O_2_Hb) (*B*), deoxygenated hemoglobin (HHb) (*C*), and total hemoglobin (tHb) (*D*). (*E*) Data processing steps from tibial NIRS signals obtained in the same participant at the supine‐to‐walk transition. Raw data (gray), low‐pass filtered (blue), and additional 5‐second moving average (red) signals are provided.

The testing protocol commenced once the devices were secured, and good signal acquisition quality was confirmed. Data were collected at 50 Hz using two instances of Oxysoft software (Artinis Medical Systems, Einsteinweg, Netherlands) running on two different laptop computers. Six time‐synchronization events were entered into both data sets simultaneously using event markers. The protocol consisted of four different postures: (i) 5‐minute baseline supine, (ii) 10‐minute ambulation, (iii) 5‐minute quiet stand, and (iv) 5‐minute supine recovery; transitions between these timed intervals are shown in Figure 2.

Other than during ambulation, participants were instructed to refrain from talking or moving. Participants were asked to walk comfortably during the 10‐minute ambulation period up and down a corridor 38 m in length, turning at each end. Timing at each turn was recorded with a stopwatch and noted for calculation of walking speed.

### Data processing

Time series O_2_Hb, HHb, and tHb data from each of the three tissue depths, as well as TSI data, from each NIRS device were imported into MATLAB R2020b (The Mathworks Inc, Natick, MA, USA), where they were time‐synchronized after calculation of the average difference between timing events entered during collection on the two respective systems. Analyses for changes in O_2_Hb, HHb, and tHb were performed on data acquired using transmitter two (Tx2, rather than Tx1 or Tx3) after assessment of tibial ultrasound images. This transmitter was chosen because its maximum penetrative depth of 17.5 mm incorporated the entire anterior cortex and medulla of the tibia, while limiting the risk of data polluted from either deeper tissues or an overrepresentation of soft tissue with the more superficial Tx1 data set. Subsequently, signals were low‐pass filtered (0.05 Hz) to remove cadence artifact and then smoothed (5‐second moving average). Comparisons between plateau values for O_2_Hb, HHb, and tHb were performed on 1‐minute averages taken from the end of each of the supine, walk, and stand periods, with values reported as the difference between these plateaus. Time to 95% of nadir values following the supine‐to‐walking transition were calculated manually. The time constant (τ) for O_2_Hb and tHb during stand was taken as the time to reach 1‐1/e of a natural‐logarithmic curve fitted to the first 120 seconds of data, starting immediately at the walk‐to‐stand transition.

Tibial depth for each participant was calculated from a longitudinal acquisition of the midline of the tibia, directly over the position at which the NIRS device was placed. Images were imported into Image J (version 1.53 k, National Institutes of Health, Bethesda, MD, USA), and an average depth from skin surface to superficial tibial cortex was calculated from three sequential measurements that were equally spaced along the length of the acquisition. Similar approach toward kinetic assessments as an investigative technique were previously reported by our laboratory for the monitoring of tissue oxygenation in exercise transitions.^(^ ^33^ ^)^

### Statistical analysis

Data sets from two participants were not included in analysis; for one participant, we could not achieve a reliable bone signal during the POST assessment despite repeated adjustment of the NIRS probe, whereas the other withdrew from the study before the POST data collection time point. All analyses were performed using R (version 4.1.2). Associations between TSI and tibial cortical depth, and TSI and hematological indices were assessed using simple regression. For assessment of changes between postures, values were averaged over the last minute of each posture and normalized to baseline supine values. Paired *t* test comparisons with Bonferroni correction were then performed at the PRE time point only, between adjacent conditions, including the baseline supine and walk, the walk and stand, the stand and end supine, and finally between the baseline supine and end supine. This statistical approach was chosen to allow for pertinent and targeted comparisons of normal physiological hemodynamic changes occurring in healthy older adults and to avoid comparing nonsequential transitions that could be influenced by prior postures or activity that would invalidate other statistical tests. Paired *t* tests were also used to compare changes in steady‐state TSI and hemoglobin concentrations for each posture transition within a given tissue between the PRE and POST time points. Differences in dynamic adaptation to changes in posture/walking (eg, TSI time to 95% nadir for supine‐to‐walk transition, and time constants for hemoglobin concentrations during walk‐to‐stand transition) were evaluated between pre‐ and post‐HDBR and between tissues using two‐way repeated measures ANOVA. Where appropriate, pairwise post hoc comparisons of interactions were corrected using Bonferroni methodology. Statistical significance is reported when *p* < 0.05. Data are reported as mean ± standard deviation.

## Results

Collections were tolerated well with no participants requesting early termination of the test. There was no difference in walking speed between time points (1.27 ± 0.13 m/s PRE versus 1.27 ± 0.15 m/s POST). Ultrasound determined depth of the superficial tibial cortex (4.6 ± 2.5 mm) and cortico‐medullary transition (11.8 ± 4.1 mm) varied among participants; however, there was no relationship between depth and TSI at either the PRE (slope −0.07, *r* ^2^ = 0.03) or POST (slope 0.145, *r* ^2^ = 0.05) time point. Similarly, TSI was not related to hemoglobin concentration (PRE slope 0.92, *r* ^2^ = 0.17 versus POST slope 0.10, *r* ^2^ < 0.01) or hematocrit (PRE slope 0, *r* ^2^ = 0.19 versus POST slope 0, *r* ^2^ < 0.01). An example time series is shown in Figure 2.

### Tissue responses after the supine‐to‐walking transition

**Fig. 3:**
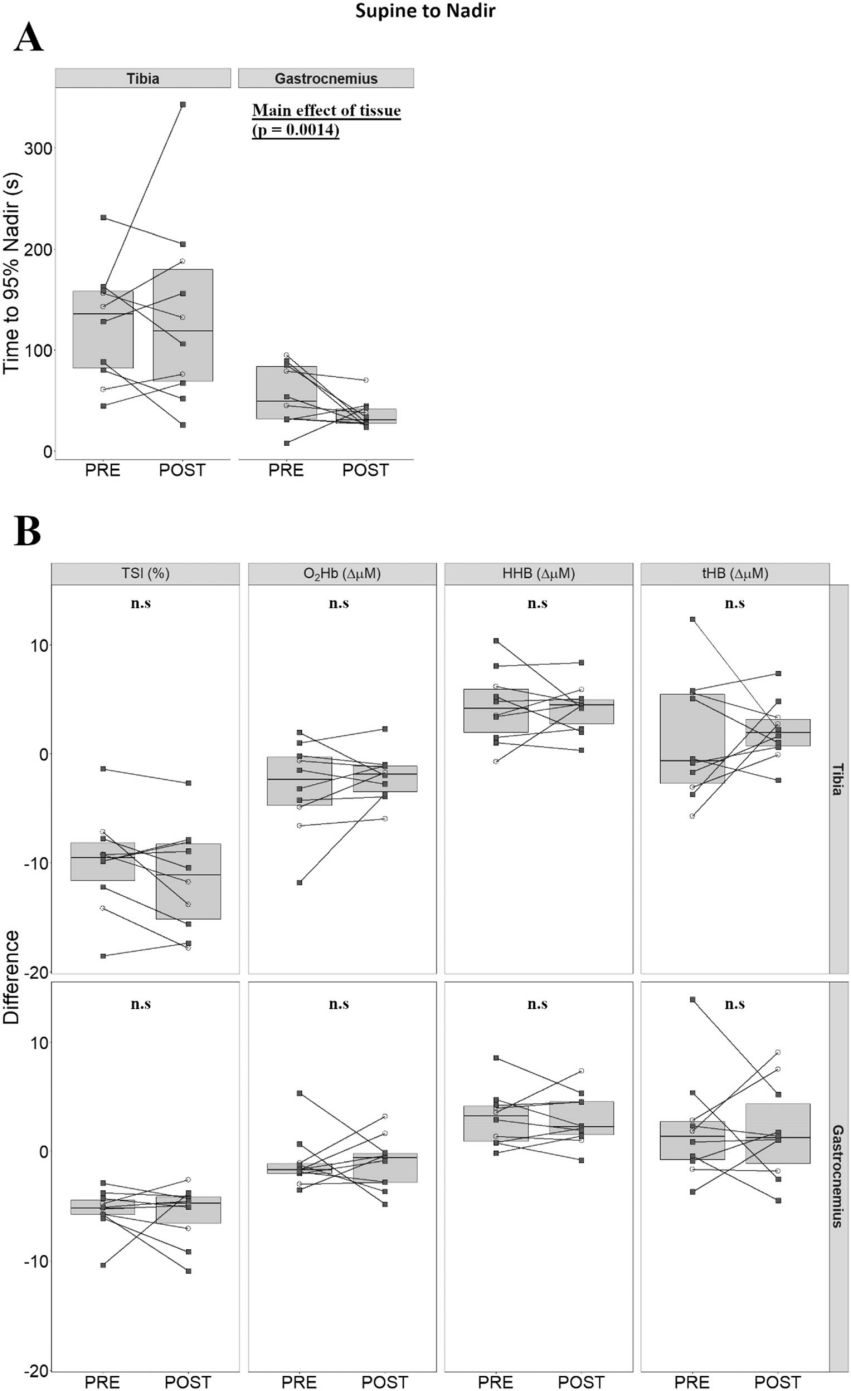
Differences between PRE and POST tibial and gastrocnemius responses, once tibial tissue saturation index (TSI) reached nadir after the start of walk (*n* = 10). Men are presented as empty circles, while women are presented as filled squares. Comparisons for time to 95% of TSI nadir values were investigated with repeated measures two‐way ANOVA (tissue * bed rest) (*A*). Differences in TSI, oxygenated hemoglobin (O_2_Hb), deoxygenated hemoglobin (HHb), and total hemoglobin (tHb) (*B*) are provided sequentially for the tibia and gastrocnemius, where statistical significance was tested for with paired *t* tests between PRE and POST time points within tissues.

TSI fell in both tissues after the commencement of walking. Time to 95% of nadir values were greater in the tibia when compared with gastrocnemius, with a main effect of tissue identified, suggesting slower kinetics at PRE (125.4 ± 56.8 s versus 55.0 ± 30.1 s, Fig. 3*A*), and similar findings at POST (135.1 ± 93.8 s versus 36.4 ± 13.9 s, tibia and gastrocnemius, respectively). At this same time point, we identified no effects of bed rest on changes in TSI, O_2_Hb, HHb, or tHb between the PRE and POST time points (Fig. 3*B*).

**Fig. 4:**
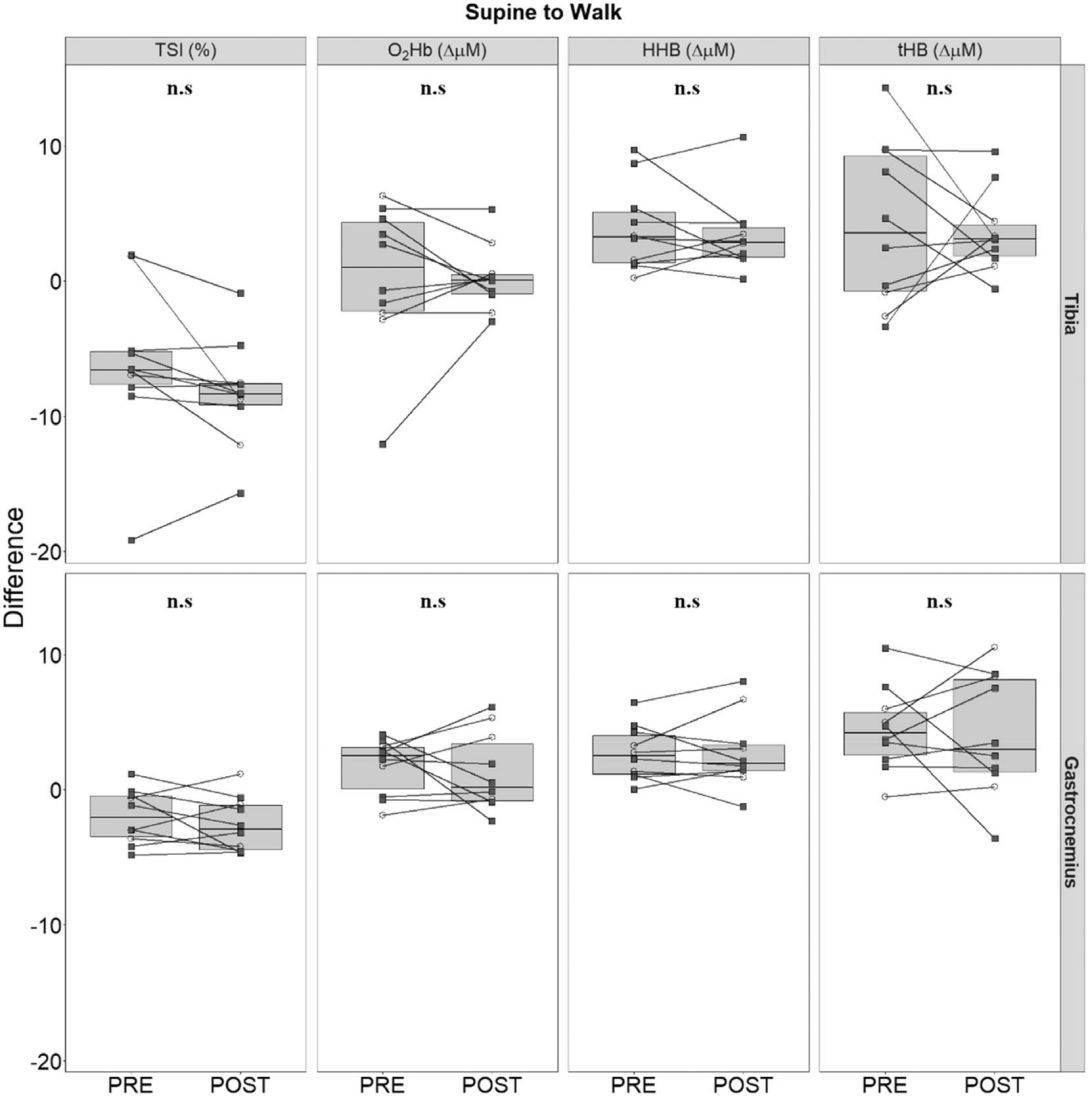
Differences between PRE and POST tibial and gastrocnemius responses when comparing the last minute of baseline supine baseline and the last minute of walking (*n* = 10). Men are presented as empty circles, while women are presented as filled squares. Differences in tissue saturation index (TSI), oxygenated hemoglobin (O_2_Hb), deoxygenated hemoglobin (HHb), and total hemoglobin (tHb) are provided sequentially for the tibia and gastrocnemius. Statistical significance was tested with paired *t* tests between PRE and POST time points within tissues.

**Fig. 5:**
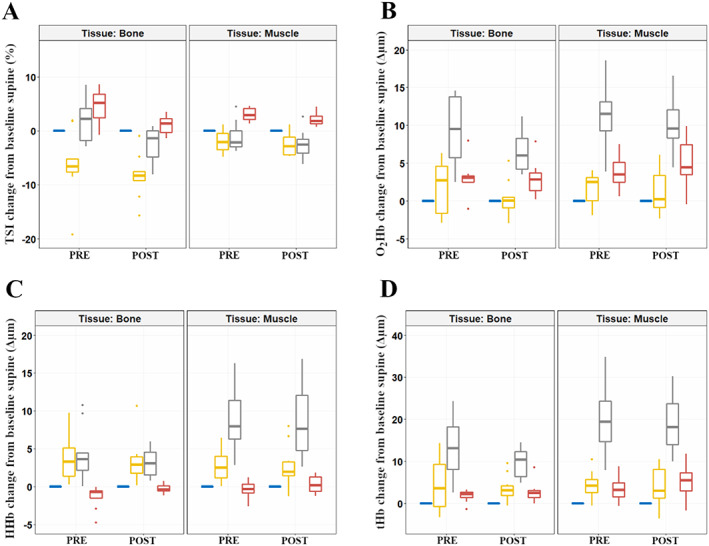
Differences in tissue saturation index (TSI) (*A*), oxygenated hemoglobin (O_2_Hb) (*B*), deoxygenated hemoglobin (HHb) (*C*) and total hemoglobin (tHb) (*D*) in the tibia (left) and gastrocnemius (right) before (PRE) and after (POST) bed rest (*n* = 10). Resting supine (blue), walking (yellow), stand (gray), and end‐supine (red) values represent the median and interquartile range (IQR) of the last minute of each posture. Table 1 provides the statistical analyses performed on the PRE bed rest results only, allowing for assessment of normal hemodynamic physiology during these changes in posture and activity (see Materials and Methods for further information why this approach was used). Effects of tissue and bed rest are further explored at each transition in Figures 2, 3, 5, and 6 with repeated measures two‐way ANOVAs.

During the last minute of walking, TSI in both tissues remained lower than supine baseline while simultaneously exhibiting greater concentrations of HHb and no change to O_2_Hb (Table 1 and Fig. 4). Although tHb increases reached statistical significance in the gastrocnemius, they did not in the tibia (Table 1). There were no effects of bed rest on changes in O_2_Hb, HHb, or tHb in either tissue (Fig. 5).

**Table 1:** Paired *t* Tests With Bonferroni Correction to Assess Hemodynamic Changes Between Sequential Experimental Transitions at the PRE Bed Rest Time Point Only, Allowing for Determination of Normal Hemodynamic Responses to Changes in Posture and During Ambulation

	Tibia				Gastrocnemius			
Transition	TSI (%)	O_2_Hb (μM)	HHb (μM)	tHb (μM)	TSI (%)	O_2_Hb (μM)	HHb (μM)	tHb (μM)
Start supine to walk	−7.4 ± 6.7 (0.027)	−0.22 ± 5.5 (n.s.)	+3.8 ± 3.1 (0.013)	+3.6 ± 6.1 (0.49)	−2.2 ± 2.1 (0.031)	+1.6 ± 2.0 (0.16)	+3.0 ± 2.1 (<0.001)	+4.5 ± 3.0 (<0.001)
Walk to stand	+9.1 ± 7.5 (0.015)	+9.2 ± 3.3 (<0.0001)	+0.6 ± 3.5 (n.s.)	+9.7 ± 3.2 (<0.0001)	+0.6 ± 1.9 (n.s.)	+9.0 ± 3.8 (<0.0001)	+6.0 ± 3.5 (<0.0001)	+15.0 ± 5.3 (<0.0001)
Stand to end supine	+2.0 ± 2.2 (0.146)	−5.8 ± 6.0 (0.119)	−5.2 ± 2.8 (<0.001)	−11.0 ± 7.6 (0.015)	+5.1 ± 2.3 (<0.001)	−6.2 ± 3.5 (0.004)	−9.8 ± 2.8 (<0.001)	−16.0 ± 7.2 (<0.001)
Start supine to end supine	+4.6 ± 2.9 (0.009)	+2.8 ± 2.5 (<0.001)	−1.5 ± 1.5 (0.113)	+1.3 ± 1.8 (0.39)	+2.9 ± 1.2 (<0.001)	+3.4 ± 2.6 (0.03)	−0.7 ± 1.5 (n.s.)	+2.7 ± 3.6 (0.338)

*Note*: Provided values represent mean ± standard deviation of deltas between average values for the last minute of each transition for tissue saturation index (TSI; %) and oxygenated hemoglobin (O_2_Hb), deoxygenated hemoglobin (HHb), and total hemoglobin (tHb) (all μM) with the *p* value in parentheses after Bonferroni correction. Values represent an additional participant (*n* = 11) that was not included in PRE to POST comparisons, as this participant completed PRE testing but did not complete POST testing.

### Tissue responses after the walk‐to‐stand transition

**Fig. 6:**
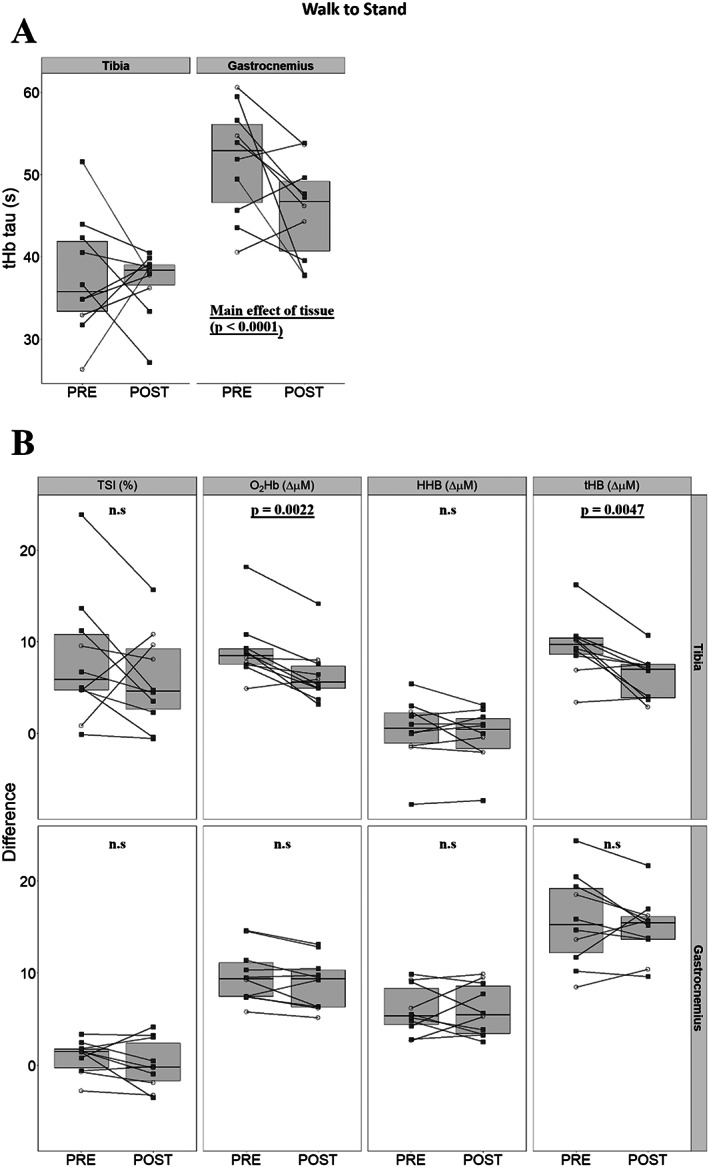
Differences between PRE and POST tibial and gastrocnemius responses when comparing the last minute of walk to the last minute of stand (*n* = 10). Men are presented as empty circles, while women are presented as filled squares. Comparisons for the tHb time‐constant (tau) were investigated with repeated measures two‐way ANOVA (tissue * bed rest) (*A*). Differences in tissue saturation index (TSI), oxygenated hemoglobin (O_2_Hb), deoxygenated hemoglobin (HHb), and total hemoglobin (tHb) (*B*) are provided sequentially for the tibia and gastrocnemius, where statistical significance was tested for with paired *t* tests between PRE and POST time points within tissues.

Comparison of the tHb time constant τ over the first 120 seconds after the walk‐to‐stand transition identified a main effect of tissue (*p* < 0.0001, Fig. 6*A*). At both PRE and POST collections, tibial tHb τ were shorter than those of gastrocnemius (PRE: 37.5 ± 7.1 s versus 51.6 ± 6.7 s, POST: 36.9 ± 4.0 s versus 45.7 ± 5.9 s, tibia versus gastrocnemius). Similar analysis of O_2_Hb kinetics identified no difference between tissues but did identify an effect of bed rest, with kinetics becoming faster at POST compared with PRE in both tissues (PRE: 44.8 ± 8.5 s versus 50.4 s ± 8.6 s, POST: 42.9 s ± 6.8 s versus 44.7 ± 6.7 s, tibia versus gastrocnemius, *p* = 0.039).

Comparing values at the end‐of‐walk to end‐of‐stand, TSI recuperated dramatically in the tibia while remaining relatively unchanged in the gastrocnemius (Table 1). In the tibia, concentration of O_2_Hb rose without increases in HHb culminating in higher levels of tHb. Conversely, in the gastrocnemius, O_2_Hb and HHb rose (Table 1). Investigating the effects of bed rest, changes in O_2_Hb plateau values (PRE: +9.2 ± 3.3, POST: +6.4 ± 3.0, *p* = 0.0022) and tHb plateau values (PRE: +9.5 ± 3.1, POST: +6.2 ± 2.3, *p* = 0.0047) at the end of stand were diminished in the tibia alone (Fig. 6*B*).

### Tissue responses after return to supine

**Fig. 7:**
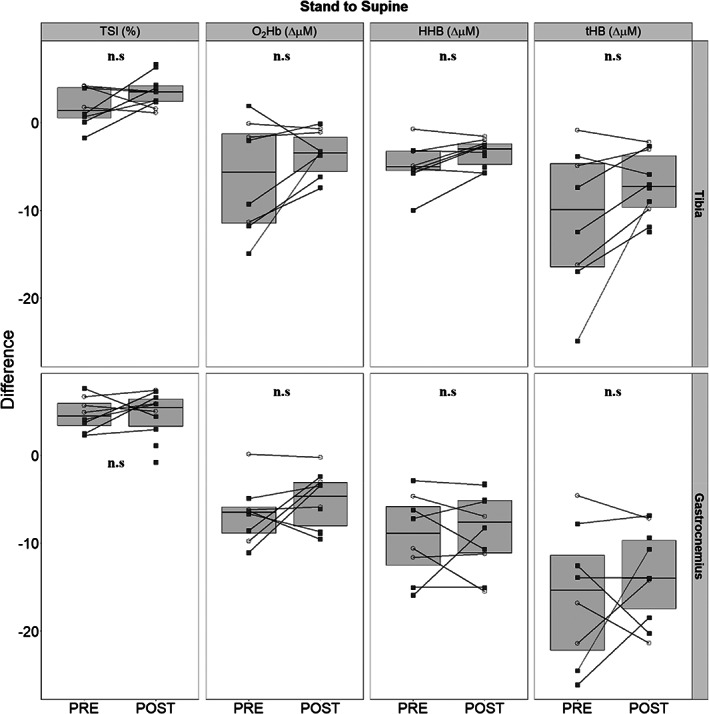
Differences between PRE and POST tibial and gastrocnemius responses when comparing the last minute of stand to the last minute of end supine (*n* = 8 PRE, *n* = 10 POST). Men are presented as empty circles, while women are presented as filled squares. Differences in tissue saturation index (TSI), oxygenated hemoglobin (O_2_Hb), deoxygenated hemoglobin (HHb), and total hemoglobin (tHb) are provided sequentially for the tibia and gastrocnemius. Statistical significance was tested with paired *t* tests between PRE and POST time points within tissues.

Return to supine led to increases in TSI with concomitant reductions to O_2_Hb, HHb, and tHb in both tibia and gastrocnemius (Table 1 and Fig. 7). Although not identifying any statistically significant changes associated with bed rest, return‐to‐supine reductions of HHb (*p* = 0.053) and tHb (*p* = 0.058) in the tibia trended toward attenuation after bed rest.

### Comparisons between start supine and end supine

Comparing baseline supine to end‐supine TSI and O_2_Hb remained slightly elevated in both tibia and gastrocnemius (Table 1). We noted a bed rest effect for TSI (PRE: +4.5 ± 2.9, POST: +1.6 ± 1.3, *p* = 0.016) in the tibia alone.

## Discussion

Our results demonstrate novel human skeletal vascular kinetics in transitions from supine rest to walking and standing and the impact of 14 days of bed rest on these kinetics. Our findings confirmed the hypothesis of tibial desaturation during walking with slower kinetics than those of the gastrocnemius, with evidence that the lower‐limb muscle pump is actively involved in venous outflow from the tibia during walking. Furthermore, we identified bed rest–induced changes to skeletal hemodynamics that may help further our understanding of mechanisms leading to bone losses during and after protracted periods of immobility.

### Tissue responses during walking

Baroreflex‐mediated sympathetic vasoconstriction of lower limb vasculature occurs during transition to upright posture, effectively reducing lower limb blood supply.^(^ ^27^ ^)^ In skeletal muscle, localized sympatholysis in the vascular beds of actively contracting muscle^(^ ^34^ ^)^ enables functional hyperemia. Vascular supply of the tibia and both heads of the gastrocnemius muscle originate at or below the popliteal artery,^(^ ^35^, ^36^ ^)^ creating parallel vascular beds in close proximity. We expected that in our participants during walking, active muscular contractions produced local vasoactive metabolites, selectively dilating muscular arterioles and reducing resistance in this vascular bed^(^ ^37^ ^)^ and increasing tissue oxygenation. In contrast, in the tibia, sympathetically‐induced vasoconstriction of feed arterioles^(^ ^30^ ^)^ would be expected to increase vascular resistance in the upright posture upon activation of the arterial baroreflex. Relative reduction in resistance in the gastrocnemius vascular bed and increase in resistance in the tibial vascular bed would, in the absence of altered perfusion pressure gradient, reduce flow to, and oxygenation of, the tibia.

At the onset of walking, time to TSI nadir in the tibia took longer than simultaneous reductions in the gastrocnemius; we saw fast dynamic compensation in the muscle that was not mirrored in the tibia. Our established penetration depth of half emitter‐receiver distance,^(^ ^38^ ^)^ or 17.5 mm given our chosen settings, likely incorporates both skeletal cells within the tibial cortex and cells within the tibial marrow, most probably adipocytes.^(^ ^39^ ^)^ The skeletal and fat tissues under examination exhibit consistent and relatively low metabolic rates when compared with the gastrocnemius muscle; rates that would not be expected to change between supine rest and either standing or walking. Consequently, and given that TSI represents the balance between oxygen supply and utilization, a fall in tibial TSI must represent reductions in oxygen delivery. Reductions in TSI noted here suggest falling vascular conductance, likely due to vasoconstriction of tibial feed vessels. The reduction to O_2_Hb and rise in HHb, without change in tHb (Table 1; Fig. 2*B–D*), support the above conclusions; the volume of blood within the tibia (tHb) did not change between supine and walking, whereas reductions to inflow resulted in relatively greater oxygen extraction, manifesting in lower O_2_Hb and reciprocal increases in HHb.

Limited investigations of marrow oxygenation in humans exist, with tissue saturations around 87.5% noted in marrow aspirates from the iliac crest of healthy volunteers,^(^ ^40^ ^)^ while mathematical models of oxygen dynamics within the bone marrow suggest very low oxygen concentrations.^(^ ^41^ ^)^ These levels are not unexpected, as a hypoxic marrow niche is crucial for the maintenance of hematopoetic stem cells.^(^ ^42^ ^)^ Despite a lack of hematopoetic activity within our tibial region of interest, it could be that maintenance of this hypoxic tendency persists after conversion to adipose marrow tissue. Our findings would indicate that periods of walking might result in repeated hypoxic exacerbation in marrow in the lower limbs and may potentially contribute to maintenance of this niche. Finally, it should be noted that this desaturation continues for at least 10 minutes of continuous walking.

### Tissue responses during quiet stand

With the transition between walking and standing, the cessation of rhythmic muscular contractions led to a sudden reduction in venous outflow from the lower limb, resulting in the rapid pooling of blood in both muscle and bone.^(^ ^43^ ^)^ Over the course of the 5‐minute stand, most of the change within the tibia can be attributed to O_2_Hb (Fig. 6*B*), with HHb showing an immediate increase (Fig. 2) but overall remaining unchanged between walking and standing (Table 1; Figs. 5 and 6*B*) as venous outflow is reestablished during standing. The large increase in tHb and O_2_Hb indicates that arterial supply transiently outstripped venous outflow in the tibia, filling it with arterial blood. Blood flow through a tissue is dependent on pressure differences between the arterial and venous vasculature in addition to vascular resistance. The lower limb muscle pump is essential for venous return against the upright hydrostatic pressure gradient,^(^ ^44^ ^)^ maintaining the pressure gradient in the musculature by aiding venous return to the heart. Although the vascular system inside the solid structure of the bone is not affected by the muscle pump, direct connection of tibial veins with veins of the gastrocnemius muscle would impose a reduced venous pressure so that the perfusion pressure gradients across the muscle and bone of the lower leg were similar. Our results, therefore, highlight the importance of the muscle pump while upright to maintain venous return from the tibia, a physiological finding that we believe has not been previously described in the literature. Finally, compared with the gastrocnemius muscle, shorter tHb τ in the tibia represented relatively faster filling, likely due to the encapsulated nature of the bone and limited compliance given reduced venous outflow.

### Tissue responses on return to supine

In both the tibia and gastrocnemius, O_2_Hb and TSI remained slightly elevated at the end of the final 5‐minute supine period when compared with baseline supine. This may in part be due to a relatively greater supply of blood to the whole lower limb after the walk and stand. Deoxygenated blood quickly left the compliant veins of the tibia and gastrocnemius as the removal of the head‐to‐foot gravitational force allowed for increased venous return from the lower limb and the consequential re‐equilibration of vascular haemodynamics.

### Hemodynamic consequences after bed rest

Hemodynamic consequences of bed rest in the long bones of the lower leg have never been investigated. Importantly, we found that increases to O_2_Hb and tHb in the tibia during standing were attenuated after bed rest, without a difference in HHb, and could therefore be indicative of reductions in arterial supply, possibly related to either changes in diameter,^(^ ^17^ ^)^ vasoactivity^(^ ^18^ ^)^ of feed vessels, or changes in hemoglobin concentration. One must remain aware that measurements of O_2_Hb, HHb, and tHb using NIRS in individuals with reduced hematocrit are unknown and may influence results at the POST collection, as losses in red cell mass and total blood volume during HDBR provokes a compensatory increase in plasma volume that results in hemodilution during the immediate post‐HDBR period.^(^ ^45^, ^46^ ^)^ Nevertheless, we provide here evidence that bed rest reduced O_2_Hb concentrations in the tibias of our participants during upright posture and that this could have resulted from reduced O_2_Hb inflow. Linking these findings to morphological changes in the tibia or to nutrient feed vessel characteristics would potentially provide important and novel mechanisms that might explain some losses of bone during bed rest, protracted sedentary behavior, and even spaceflight.

### Limitations

NIRS devices do not allow for direct measurements of quantified blood flow or O_2_ utilization in a tissue; rather, we infer these variables from measurements of changing hemoglobin concentrations in the different tissues. We acknowledge that the precise behavior of near‐infrared light in bone tissue is not as well characterized as muscle, with a very limited literature base to support our assumptions for differential pathlength factor and scatter properties of the tibia. However, Draghici and colleagues^(^ ^26^ ^)^ recently used NIRS to investigate hemoglobin content changes at the same location on the tibia as the present study and used Monte Carlo simulations to examine how differential pathlength factor changes as a function of wavelength and different distances between the light source and detector. We extrapolated Draghici and colleagues’^(^ ^26^ ^)^ findings to estimate plausible settings that could be used with our device, which has a larger distance between between the lightsource and detector. Nevertheless, comparisons of differences in the magnitude of change with walking or posture change between bone and muscle tissues could be influenced by the selected bone differential pathlength factor for the relative concentrations of O_2_Hb, HHb, and tHb, or bone scatter coefficient for TSI. Accordingly, in our analysis, we only performed comparisons between bone and muscle tissues for their dynamic responses (eg, TSI time to 95% nadir in the supine‐to‐walk transition, or time constant for tHb during the walk‐to‐stand transition), as the kinetics variables for the different tissues are independent of the signal shifts or changes in amplitude that occur when different differential pathlength factors or scatter coefficients are assumed. Only within‐tissue comparisons were performed to assess the affects of posture/walking or HDBR on steady‐state changes in TSI, as well as O_2_Hb, HHb, and tHb concentrations in the bone or muscle, which prevented potentially erroneously concluding that larger or smaller amplitude changes may occur in different tissues. Importantly, it is not expected that the behavior of light in either the bone or muscle of a given individual at the same anatomical location dramatically changes in ~3 weeks of time (ie, time between pre‐ and post‐HDBR assessments in the present study), enabling the effects of HDBR to be evaluated on a given tissue when the same NIRS settings were used in pre‐ and post‐HDBR assessments.

With using a continuous‐wave NIRS device, we were unable to measure absolute concentrations of O_2_Hb, HHb, or tHb in each tissue, but instead could only measure changes in concentration within each tissue for a given posture or exercise transition. Accordingly, measured changes in hemoglobin variables do not account for potentially different initial hemoglobin concentrations. Investigations with frequency or time‐resolved NIRS devices may provide more quantifiable measurements of these variables. Despite the well‐established penetration depth of NIRS in soft tissue,^(^ ^38^ ^)^ we cannot be certain of penetration depth of NIRS devices in the tibial bone. However, given the previously established and reproducible results within the tibial tissue and well‐documented ability to record brain oxygenation accurately across the skull, we believe these signals do represent hemodynamics within the tibia. Finally, we reported data from the middle transmitter (Tx2) because it was deemed most representative of the tibial bone after considerations of tissue depth and between‐participant characteristics. This choice allowed us to refine measurements of O_2_Hb, HHb, and tHb; however, measurement of TSI utilized data from all three sensors to apply the spatially resolved spectroscopy technique.^(^ ^47^ ^)^ Therefore, tibial TSI incorporates minimal amounts of subcutaneous adipose tissue, but the vast majority will be of bone origin, and the similarity of responses between participants despite considerable differences in subcutaneous adipose tissue thickness reaffirms the ability of these devices to measure signals from the superficial anteriomedial tibia.

The 14‐day HDBR study at McGill University Health Centre enrolled a total of 23 participants over the course of 6 months and four cohorts. However, our NIRS investigations could only include participants during the last two cohorts, so numbers of participants were limited. Therefore, we were unable to investigate for differences between exercise and control participants or between men and women. However, treating the 10 participants as one experimental cohort still allowed for identification of bed rest effects on skeletal hemodynamics. It should be noted that our findings of altered tibial hemodynamics occurred despite most of our cohort performing routine cycling and resistance exercise. Unfortunately, we did not have access to simultaneous measurements of morphological or densitometry data from the tibia to correlate our findings of altered TSI, O_2_Hb, HHb and tHb to localized bone changes. Therefore, we cannot determine the extent to which these changes in vascular kinetics might relate to established skeletal consequences of bed rest.

In conclusion, the ease and tolerability of these data acquisitions should allow for investigation of complex kinetic comparisons between tibia and muscle in the future, thereby providing invaluable assessment of human skeletal vascular kinetics and furthering our understanding of vascular and skeletal relationships. Our finding of protracted tibial desaturation during ambulation is an important and undescribed phenomenon providing evidence of a potential exacerbation of a relative hypoxia within these tissues. Identification of rapid increase in O_2_Hb with consequent tHb filling in the tibia following the walk‐to‐stand transition indicates the active role of lower‐limb muscle pumping for maintenance of venous outflow from the tibia. After HDBR, the reduced O_2_Hb concentrations while standing with potential concomitant attenuation of post‐ambulation TSI recovery could indicate altered tibial arterial supply.

## Author Contributions

**Eric T Hedge:** Conceptualization; data curation; formal analysis; investigation; methodology; writing – review and editing. **Laurence Vico:** Writing – review and editing. **Richard L Hughson:** Formal analysis; funding acquisition; resources; supervision; writing – review and editing. **Carmelo J Mastrandrea:** Conceptualization; data curation; formal analysis; investigation; methodology; project administration; validation; visualization; writing – original draft; writing – review and editing.

## Disclosures

The authors disclose no conflicts of interest.

### Peer Review

The peer review history for this article is available at https://www.webofscience.com/api/gateway/wos/peer-review/10.1002/jbm4.10756.

## Data Availability

The data that support the findings of this study are available from the corresponding author upon reasonable request.
